# Fasa Registry on Acute Myocardial Infarction (FaRMI): Feasibility Study and Pilot Phase Results

**DOI:** 10.1371/journal.pone.0167579

**Published:** 2016-12-01

**Authors:** Ehsan Bahramali, Alireza Askari, Habib Zakeri, Mojtaba Farjam, Azizallah Dehghan, Kazem Zendehdel

**Affiliations:** 1 Department of Cardiology, Fasa University of Medical Sciences, Fasa, Iran; 2 Noncommunicable Diseases Research Center, Fasa University of Medical Sciences, Fasa, Iran; 3 Faculty of Medicine, Shiraz University of Medical Sciences, Shiraz, Iran; 4 Department of Medical Pharmacology, School of Medicine, Fasa University of Medical Sciences, Fasa, Iran; 5 Cancer Research Center, Cancer Institute of Iran, Tehran University of Medical Sciences, Tehran, Iran; Azienda Ospedaliero Universitaria Careggi, ITALY

## Abstract

**Background:**

Myocardial infarction (MI) is the leading cause of death in Iran. Every attempt to improve treatment patterns and patient outcomes needs a surveillance system to both consider the efficacy and safety measures. Fasa Registry on Myocardial Infarction (FaRMI) is the first population-based registry for acute MI in Iran targeted to provide meticulous description of patients’ characteristics, to explore the management patterns of these patients, to discover the degree of adherence to the practice guidelines, and to investigate the determinants of poor in-hospital and later outcomes.

**Methods:**

A diagnosis of acute MI (type I, II and III) was made upon the accepted criteria by the attending cardiologists and types IV and V MI were excluded. Two registrar nurses gathered data on demographics, place of residence and ethnicity, past medical history, risk factors, and the clinical course. Management patterns in the pre-hospital setting, during the hospital stay and at the discharge time were recorded. Routine laboratory results and cardiac biomarkers on three consecutive days were registered.

**Results:**

pilot phase included the first 95 patients, 63.5% of whom were men and 31.5% were women. With a mean age of 62.89±13.75 years among participants, the rate of premature MI was 31.8%. ST segment elevation MI accounted for 68.2% cases and inferior wall was the most prevalent region involved followed by anterior and posterior walls.

**Discussion:**

Obtained data on the characteristics of patients suffering an MI event revealed the major determinants of delay in initiation of therapies and contributors of poor outcome. Completeness of data was guaranteed upon involvement of multiple checkpoints and data quality was secured by means of automatic validation processes in addition to weekly physicians’ roundups.

**Conclusion:**

Execution of FaRMI in the form presented is feasible and it will build up a comprehensive population-based registry for MI in the region.

## Introduction

Ischemic heart disease (IHD) is the leading cause of death worldwide [1]. In Iran, data from 50045 participants of Golestan cohort study with a total of 234928 person years of follow-up (median 4.7 years per participant) revealed that half of all deaths (1073 out of 2145) were because of circulatory causes including 624 deaths from IHD [2]. Carrying the highest mortality rates worldwide and in Iran, CV diseases require special approaches for systematic documentation of incidence rates and changing trends over time. This is also a mandatory pre-requisite for surveillance purposes and monitoring for national strategies to reduce CV risk. Moreover, based on the local health policies and availability of regional health services, natural history and management of MI vary largely across time and place.

A registry system will best serve for the intentions of identification and control of the major determinants of MI mortality and morbidity. International Global Registry of Acute Coronary Events (GRACE), the Euro Heart Survey program, French Registry of acute MI (FAST-MI) or British Myocardial Ischemia National Audit Project (MINAP) were successful examples of MI registries worldwide with their findings constituting our current knowledge and being implemented as routine practice guidelines [3–6]. They’ve also witnessed a decline in the CV mortality rate worldwide over the last decade which is attributed both to improved primary prevention and management of CV diseases [7]. In the Middle East however, although this trend is apparently present, the details of CV outcome measures remained elusive and need to be described.

While a functioning MI registry system minding the balance between the necessary data compliment along the logistic, laboratory and budget shortages in Iran is lacking, evidence for the quality of care and the degree of adherence to the international guidelines in management of acute MI is consequently missing. Furthermore, national guidelines have not been developed to consider local and logistic limitations and make definitions of the standard of care. The previous attempts to establish MI registries in Iran shared several shortcomings: there are no information on missing cases and the completeness of the registries; indexing the MI events were not accurate because it has not been carried out by the trained personnel; physicians were reluctant in documenting the diagnosis and treatment pathways; a timely and thorough laboratory assessment of cardiac enzymes has not been carried out due to logistic restrictions, and there were no dedicated medical personnel as registrars and usually the in-charge for registry was overwhelmed with other responsibilities in the hospitals.

With these serious limitations to a precise and well operated MI registry in Iran and to fulfill the multiple purposes of identifying treatment patterns (institution and physician oriented), promoting time-to-treatment and other quality improvements, as well as gathering real-world efficacy (both clinical and financial) and safety data, Fasa Registry of MI (FaRMI) was designed in 2015 to systematically enroll patients with a diagnosis of acute MI. With a well-operated death registry in the region, we would be able to identify deceased patients with acute MI as well thus promoting FaRMI to a population-based registry for MI.

## Methods

### Objectives

To provide meticulous description of patients’ characteristics admitted with acute MI in Fasa, to explore the management patterns of these patients, to discover the implementation of practice guidelines in a typical Iranian university hospital, and to investigate the determinants of poor in-hospital and later outcomes. As this is a population based registry with monthly regional death registry reviews and verbal autopsies per needed, determination of acute MI incidence density rate and prevalence will be made. Other objectives were to assess correlations between ethnicity, place of residence, socioeconomic status, depression, stressful life events, and nutrition and MI outcomes. Other FaRMI aims were to define the best pre-hospital as well as in-hospital management strategies to improve outcomes, to determine the relationships between biomarkers and morbidity-mortality after acute MI and to build a structured database for further research. Comparisons between various disciplines across the country is now possible based on the availability of similar data in other regions. FaRMI is credited with the first approved national MI registry system in the ministry of health (MOH) center for registries and for the first time in Iran will enable documentation of the long term (5 years) outcomes of patients presenting with acute MI in 2020.

The study is carried out by the cardiology department and Noncommunicable Diseases (NCD) research center at Fasa University of Medical Sciences (FUMS) and is funded by Ministry of Health national center for disease registries grant number 1290007/10516 for 5 years. Dedicated research projects within the main study are granted by FUMS and NCD research center awarded complementary grants.

Six cardiologists including three interventionists participated in the registry. They have concurred to adhere to their typical therapeutic approach and were expected to avoid modifications.

Each enrollee was informed by the registrar nurse about the study and signed a written consent. In case of patients passing away before signing the consent, patient’s next of kin was asked for the permission of retaining the data.

Study protocol was reviewed and approved by the Ethics Committee for Biomedical Research at FUMS (code: E-9310) and data recording and handling met the regulations provided by the Iranian communication law.

### Patients

Every patient admitted in an intensive care unit (ICU) or cardiology department with a diagnosis of acute MI characterized by elevation of serum cardiac troponin I or creatinine kinase—muscle brain type (CK-MB) levels associated with at least one of the following:

- symptoms compatible with myocardial ischemia,
- development of new pathologic Q waves,
- ST-T changes compatible with myocardial ischemia,

has been enrolled type (I and II) [8]. Besides patients in other hospital wards were monitored daily for an elevated troponin level in the hospital integrated electronic system by the registrar nurse. After consulting with the cardiologist and confirmation for an acute MI upon the mentioned criteria, enrollment eventuated. ST segment elevation myocardial infarction (STEMI) was diagnosed when ST elevation > 1 mm was present in at least two contiguous leads in the presenting ECG, or when presumed new left bundle branch block (LBBB) was observed. A diagnosis of non-STEMI (NSTEMI) was made in the absence of STEMI and the presence of the inclusion criteria. Cardiac death with symptoms suggestive of myocardial ischemia and presumed new ischemic changes on the ECG or new LBBB but before cardiac biomarkers were determined or before cardiac marker values would be increased was considered as acute MI too (type III) [8].

Patients with unstable angina, iatrogenic MI immediately following PCI or CABGs (types IV and V), and presence of an alternative diagnosis against acute MI such as acute myocarditis or worsening heart failure based on the cardiologist’s interpretation were excluded.

### Description of Fasa and its health network

Fasa university hospital is the only receiving institution with full facilities including chest pain units, CCU-ICUs, catheterization lab and cardiac surgery operating rooms to serve MI patients in the region. It is referral for a population of 220000 people residing in one major city and 34 surrounding towns and villages. Approximately 8 centers could receive MI patients at their emergency rooms, but virtually all of them referred these patients to Fasa university hospital. Patients were enrolled in FaRMI from more distant (70–200 km) towns as well when they were transferred to Fasa university hospital for primary or rescue PCI. Urban and rural family physician program have been previously implemented in the region for the past decade and the primary health care system consisting of healthcare workers (Behvarz) and health houses in towns and villages date back here to more than 30 years ago.

### Recruitment

Two dedicated registrar nurses were trained by the principal investigator cardiologist in three months and they were in charge for all the enrollments. The attending cardiologist who visits all the patients everyday including holidays, is responsible to label acute MI cases. Patients are recruited consecutively by the registrar nurses everyday except for holidays and data are recorded electronically. They fill in a computerized case record form using an android tablet online and data are recorded simultaneously in a server computer in the university network with reliable firewalls. Hospital records are reviewed for all eligible patients; moreover, additional data are obtained using dedicated questionnaires and forms by registrar nurses. For the missing information, the principal investigator cardiologist is available for consultation and data are obtained well timed.

### The data

Demographic data, place of residence and ethnicity, past medical history, risk factors, and clinical course including presenting symptoms, symptoms onset time, admission Killip class, management in the pre-hospital setting, during the hospital stay and at discharge were recorded. Routine laboratory results on admission and cardiac biomarkers on three consecutive days were registered. A list of all in hospital complications including cardiogenic shock, left ventricular (LV) systolic dysfunction, heart failure with associated Killip class, arrhythmias, renal failure, stroke, and major bleeding was filled in by the attending cardiologist. LV ejection fraction obtained by echocardiographic eye balling method was recorded when assessed at any time during the hospital stay. Blood samples were collected from arterial access for the patients undergoing coronary angiography during their hospital stay and a bio bank with a total of 9 cc of frozen serum, plasma, whole blood and Buffy coat samples was compiled.

### Patients’ follow up

Registrar nurses receive alerts on the homepage of FaRMI software after logging in everyday, showing them a list of participants with due follow up periods. One, 3 and 12 months after discharge they contact patients or their families and asks for any occurred hard or soft events for the past follow up months. With specific focus on recording the occurrence of myocardial revascularization, hospitalization for heart failure, stroke and bleeding events, registrar nurses interview all the patients or their close relatives via telephone. For each reported event leading to hospitalization or death, hospital reports are sought and analyzed by a team of physicians supervised by the principal investigator cardiologist. Three internal medicine residents review hospital records separately to reach the diagnosis and in case of any discrepancies, a final decision is reached upon group discussions. Regional and national death registers are searched for the cause of death and in case of nonsense coding, verbal autopsy is made by trained personnel.

### Data quality

All data fields are sensitive to outliers and missing values. The registrar nurse is notified automatically before saving forms with empty or unusual field values. A team of research assistants help monitoring the data weekly and verify the entries. Central database that contains all the information gathered is analyzed by the steering committee every month and completeness of data is assured accordingly.

### Availability of data

Study protocol and questionnaires are available online at ncdrc.fums.ac.ir and individual researchers and institutes can ask for data after submitting their research summary protocol to the online submission tool provided under the collaboration tab at ncdrc.fums.ac.ir. Steering committee of NCDRC will decide on data release and welcome international collaborations. FaRMI is linked to the PERSIAN cohort database, which has enrolled nearly 10000 participants in a prospective population based cohort study in the region. It serves as an outcome measurement tool and notifies the executive team by email when common national codes, which are unique to every individual in the country, are registered.

### Statistical methods

Continuous data are reported using mean and standard deviation with the, minimum and maximum depending on the normality assumptions. Discrete variables are reported with counts and percentages.

### Study progress

The first patient was enrolled in December 2014 and enrollment is anticipated to continue through 2020. As of June 27, 2016, 421 patients have been enrolled. Pilot phase included the first 95 patients, data for whom was discarded after analysis because of the learning curve of the engaging staff as well as the applied software updates.

### Strengths and weaknesses

Presence of a medical university regionally responsible for well being of the population, improving health indices and educating medical personnel, provides an integrated network that allows timely and easy data acquisition from different health sectors. The main strength of the present registry is that a detailed data acquisition form including overall 317 fields for each patient has to be filled in with recruitment of two dedicated trained nurses and under direct supervision of two cardiologists, two general practitioners and a team of research assistants. Extending patient enrollment to villages and small towns with previously well-established health networks that perfectly record every event in their handled population, besides reviewing death certificates monthly make it possible to build a population based registry for acute MI. As the main medical facility serving acute MI patients, Fasa university hospital was the only enrolling hospital and all the attending cardiologists are cooperative in the registry. Therefore, a diagnosis of acute MI is made by the professionals with very high precision. Disclosure of the practice patterns and patient outcomes are banned generally except for the providing cardiologist and this helps that caregivers do not hesitate to participate or modify their routines. Although it is unlikely that a person suffering from acute MI do not seek medical care, there are rare instances that she/he survives a non-fatal MI thus not being enrolled in FaRMI. Still more frequently people suffer an acute MI event outside FUMS territory therefore FaRMI cannot claim to represent a comprehensive population based MI registry. Of note some are captured after returning to their local health network by monthly reviews of the outcome charts in health houses. For patients outside cardiology wards, a process of daily inspection of the hospital laboratory reporting system is rendered by the registrar nurse. However, cardiac biomarker assessments are only performed per physician’s order. So an acute MI patient with atypical or overlapping symptoms can be missed in a non-cardiology ward if no cardiac biomarkers were obtained. The interpretation of MI incidence density rate, natural history and clinical outcomes in FaRMI must be viewed in this context, considering sources of bias. The prepared bio bank only has collected samples of patients during coronary catheterization and blood is not necessarily taken at admission. Therefore, future research must take this into account in that criteria of research protocols requiring on admission samples are not met.

## Results

Pilot phase included the first 95 patients with acute MI. Patients were labeled by the full time resident attending cardiologist initially; alternatively, the registrar nurses screened patients for presence of inclusion criteria. Among the initially screened population, 10 patients were excluded eventually because they had at least one exclusion criteria. Four patients had an alternative diagnosis, 3 had inconclusive death certificates and 3 were diagnosed with iatrogenic acute MI after cardiologist round up. Two of the enrollees were captured from a non-cardiology ward among a total of 29 screened patients during the pilot phase. Finally, 85 cases including 54 (63.5%) male and 31.5 (36.5%) female participants entered the pilot study analysis. Mean of their age was 62.8 ± 13.7, with a minimum and maximum of 30 and 96 years respectively. Premature MI, defined as MI event before 55 years of age accounted for 31.8% of the cases whereas 49.4% were between 55–70 and 18.8% were over 70 years of age. Fifty-three (62.4%) of enrollees were urban habitants and 32 (37.7%) were from rural areas. Twenty-three (27.1%) patients were brought to hospital by an ambulance with an ambulance time (time from getting on the ambulance to enter the hospital triage) of 38 ± 14 minutes. There was no difference between the ambulance time for STEMI and NSTEMI (31 ± 7 minutes versus 39 ± 16 minutes respectively; p = 0.41). The transfer rate which is the percentage of patients referred to another tertiary hospital in the nearby city (Shiraz), 150 km west of Fasa, is 11.9%. The primary reason for transfer was the transient shutdown of the catheterization laboratory during the enrollment for the pilot study. The most common presenting symptoms of acute MI are presented in Table 1. For patients reported to have inconclusive symptoms at the initial presentation, 11 were diagnosed with acute MI only after a typical rise in serum troponin levels and 31 were screened because of the ECG changes compatible with acute MI. Fifty-eight (68.2%) patients experienced STEMI and 27 (32.2%) NSTEMI. The location of acute MI in STEMI patients is presented in Table 2. One patient with old LBBB pattern as well as the two with unknown new or old LBBB were labeled as NSTEMI after fulfilling the inclusion criteria.

**Table 1:** The frequency of myocardial infarction symptoms

	Yes frequency (%)	No frequency (%)
Chest pain	68 (80.0)	17(20.0)
Breathlessness	30 (35.3)	55 (64.7)
Diaphoresis	15 (17.6)	70 (72.4)
Syncope	2 (2.4)	83 (97.6)
Back pain	1 (1.2)	84 (98.8)
Epigastria pain	17 (20.0)	68 (80.0)
Jaw pain	8 (9.4)	77 (90.6)
Shoulder pain	31 (36.5)	54 (63.5)
Cold sweating	49 (57.6)	36 (42.4)

**Table 2:** The location of AMI in STEMI patients

One region involved	Frequency (%)	Two regions involved	Frequency (%)
Anterior	25 (30.48)	Inferior-posterior	5 (6.09)
Inferior	31 (37.80)	Anterolateral	4 (4.87)
Lateral	1 (1.22)	Posterolateral	2 (2.43)
Posterior	0 (0)	Inferior-RV	2 (2.43)
Isolated RV	0 (0)	Inferior-lateral	1 (1.22)
New LBBB	0 (0)	Anterior-posterior	1 (1.22)
		Lateral-RV	1 (1.22)
		Anterior-inferior	1 (1.22)

RV: right ventricle, LBBB: left bundle branch block

Risk factors: Mean height and weight were 164 ± 10 cm, 64.0 ± 11.9 kilograms respectively. Thirty-one (36.5%) of them were current smokers and 4 (4.7%) were ex-smokers. Two (2.4%) used water pipe regularly and 4 (4.7%) consumed opium. Forty-five (52.9%) patients had a history of hypertension, 16 (18.8%) and 21 (25%) were known cases of diabetes mellitus and hyperlipidemia respectively.

Cardiopulmonary resuscitation (CPR) was necessary for three of the patients in the ambulance and two received electrical cardioversion. Seven patients underwent CPR in the hospital and three received electrical cardioversion. Five (5.9%) died, 2 (2.4%) in emergency department and 3 (3.6%) in hospital wards.

Reliable determination of LVEF was achieved in 87% of patient where 10.8% had sever systolic dysfunction (LVEF<35%), 60.8% has moderate systolic dysfunction (35≤LVEF<50) and 28.4% had preserved or normal systolic function (LVEF≥50). Recorded complications were as follow: 7.1% experienced cardiogenic shock; 36.4% were diagnosed with heart failure Killip class II, III or IV; 4.7% experienced re-infarction after rise in cardiac enzymes and 9.4% developed ventricular arrhythmias including sustained ventricular tachycardia and ventricular fibrillation.

Regarding the medications administered, 82.4% received 325 mg loading dose of acetyl salicylic acid (ASA), 16.5% didn’t receive ASA because of a contraindication and 1.2% didn’t receive ASA for unknown reasons; 84.7% received Clopidogrel stat dose; 45.9% received the stat dose of a beta blocker where only 7.1% had a contraindication to beta blocker use and the remaining 47% of patients didn’t receive it because of unknown reasons. Only three patients with STEMI hadn’t have a reperfusion strategy and among the 94.8% who received a reperfusion therapy, 65.5% received streptokinase and 34.5% underwent primary PCI.

The mean hospitalization duration was 6.2 ± 8.3 (range: 1–64) days and the median was 4 days.

## Discussion

FaRMI provides real world insights toward the detailed characteristics of patients with STEMI and NSTEMI and at the same time documents the practice patterns and MI outcomes in the region for the first time. As the coverage of primary PCI as the management goal of STEMI is increasing in the country, subjective measures for a timely treatment and outcomes are compiling which allows the health policy makers to better evaluate the costly procedure. Enrollments of patients were fulfilled with excellent precision and a cardiologist approved each diagnoses. Missing data were kept at a minimum and in the pilot phase, 98 percent of the patients had at least one, 96 percent have two and 73 percent had three serum troponin levels measured. In-hospital mortality rate was 8.2% which is less than the reported rates in most countries [9–11]. It may reflect that the fatal cases of MI didn’t reach the hospital and can be attributable to the under-development of primary care facilities receiving MI patients. However, this needs further research to investigate for the other possible explanations for this observation. Type I, II, IV and V acute MI were readily diagnosed as the attending cardiologists have been indexing the events. In the pilot phase we have identified three iatrogenic cases (two after CABG and one post PCI) and the registrar nurses were trained as well to discriminate the different acute MI types. For the two cases of early death upon arrival to the hospital (type III acute MI), we didn’t have serum troponin levels measured and a diagnosis of death due to acute MI was made upon the cardiologist’s discretion. Besides with the current hospital settings, it was a usual scenario that an event of death occurred early on arrival to the hospital, especially late at night, when the attending cardiologist was not sought. This possible case then did not receive cardiologist’s expert opinion and thereby a diagnosis of type III acute MI was missing. This pilot phase experience led to the process that the registrar nurses look for such mortalities over night each day and consult with the cardiologists for a possible acute MI as the cause of death.

Death certificates in rural areas had several shortcomings as well. Health care workers (Behvarz) reported their mortality report only upon request and there were no systematic death reporting system for those without a valid death certificate. We also noticed that their verbal autopsies were not flawless and needed to be validated. As a result, a parallel study to validate the verbal autopsies used by the health care workers were accomplished which showed that the questionnaires has sufficient sensitivity and specificity (87.5 and 99.4 percent respectively) to detect MI as the cause of death (unpublished data).

## Conclusion

The population based registration of acute MI cases in Fasa was feasible under the above mentioned framework and the results of the pilot phase are promising. Ongoing death registrations by health care workers in rural areas needs continuous surveillance to achieve to a valid population based MI registry.
